# Integrated analysis of two-lncRNA signature as a potential prognostic biomarker in cervical cancer: a study based on public database

**DOI:** 10.7717/peerj.6761

**Published:** 2019-04-22

**Authors:** Wenjuan Wu, Jing Sui, Tong Liu, Sheng Yang, Siyi Xu, Man Zhang, Shaoping Huang, Lihong Yin, Yuepu Pu, Geyu Liang

**Affiliations:** 1 Key Laboratory of Environmental Medicine Engineering, Ministry of Education, School of Public Health, Southeast University, Nanjing, Jiangsu, China; 2 Department of Medical Insurance, School of Public Health, Southeast University, Nanjing, Jiangsu, China; 3 Department of Histology and Embryology, Medical School, Southeast University, Nanjing, Jiangsu, China

**Keywords:** Long non-coding RNAs, Cervical cancer, Survival, Prognostic, Biomarkers

## Abstract

**Background:**

Cervical cancer (CC) is a common gynecological malignancy in women worldwide. Evidence suggests that long non-coding RNAs (lncRNAs) can be used as biomarkers in patients with CC. However, prognostic biomarkers for CC are still lacking. The aim of our study was to find lncRNA biomarkers which are able to predict prognosis in CC based on the data from The Cancer Genome Atlas (TCGA).

**Methods:**

The patients were divided into three groups according to FIGO stage. Differentially expressed lncRNAs were identified in CC tissue compared to adjacent normal tissues based on a fold change >2 and <0.5 at *P* < 0.05 for up- and downregulated lncRNA, respectively. The relationship between survival outcome and lncRNA expression was assessed with univariate and multivariate Cox proportional hazards regression analysis. We constructed a risk score as a method to evaluate prognosis. We used receiver operating characteristic (ROC) curve and the area under curve (AUC) analyses to assess the diagnostic value of a two-lncRNA signature. We detected the expression levels of the two lncRNAs in 31 pairs of newly diagnosed CC specimens and paired adjacent non-cancerous tissue specimens, and also in CC cell lines. Finally, the results were statistically compared using *t*-tests.

**Results:**

In total, 289 RNA sequencing profiles and accompanying clinical data were obtained. We identified 49 differentially expressed lncRNAs, of which two related to overall survival (OS) in CC patients. These two lncRNAs (ILF3-AS1 and RASA4CP) were found together as a single prognostic signature. Meanwhile, the prognosis of patients with low-risk CC was better and positively correlated with OS (*P* < 0.001). Further analysis showed that the combined two-lncRNA expression signature could be used as an independent biomarker to evaluate the prognosis in CC. qRT-PCR results were consistent with TCGA, confirming downregulated expression of both lncRNAs. Furthermore, upon ROC curve analysis, the AUC of the combined lncRNAs was greater than that of the single lncRNAs alone (0.723 vs 0.704 and 0.685), respectively; *P* < 0.05.

**Conclusions:**

Our study showed that the two-lncRNA signature of ILF3-AS1 and RASA4CP can be used as an independent biomarker for the prognosis of CC, based on bioinformatic analysis.

## Introduction

Cervical cancer (CC) is still a serious public health problem worldwide. Although there have been advances in screening and early diagnostic methods in recent years (Leyden et al., 2005), more than 85% of cases and deaths are in the developing countries (Jemal et al., 2011). The 5-year survival rate of advanced-stage patients also remains below 40% and the poor prognosis of CC is a serious problem facing these patients (O’Mara, Zhao & Spurdle, 2016). Early diagnosis and prediction of outcomes is an effective method to improve the prognosis of CC. However, molecular markers that can predict the prognosis of CC remain unknown. Thus, there is an urgent need to identify effective biomarkers that predict the survival of patients with CC.

Long non-coding RNAs (lncRNAs) are non-coding RNAs with a length of more than 200 nucleotides, which are widely present in the genome and can regulate gene expression (Cao et al., 2016; Guo et al., 2015; Mercer, Dinger & Mattick, 2009). They are involved in the development of various cancers and diseases. Increasing evidence suggests that different cancer types have different lncRNAs differentially expressed in tumor tissue (Wang et al., 2016; Zheng, Hu & Li, 2016), including CC (Hosseini et al., 2017). At present, many studies have reported that lncRNAs can be used as biomarkers for the diagnosis of CC. For example, lncRNA expression has been characterized in early CC (Shang et al., 2016), and the lncRNA RNAPVT1 may be a novel biomarker for early noninvasive diagnosis of CC (Yang et al., 2016a). However, only a few studies have reported lncRNAs as biomarkers for overall survival (OS) of CC.

The Cancer Genome Atlas (TCGA) database has provided an application platform of large-sample-size genome sequencing data for CC. Although a number of lncRNAs have been characterized and predict clinical diagnosis in CC, there are still conflicting results from previous studies. In the present study, lncRNA differential expression profiles were obtained and combined with clinical features from the TCGA database. We identified lncRNAs differentially expressed between CC tissues and normal cervical tissues by analyzing the high-throughput lncRNA sequencing data which were downloaded from TCGA database. In addition, we investigated the prognostic value of the differentially expressed lncRNAs. Through further analysis, we found a two-lncRNA signature that can effectively predict the prognosis of CC patients. This may lead to new therapeutic interventions in CC.

## Materials and Methods

### The Cancer Genome Atlas database and samples

RNA sequencing data from 307 cases of cervical squamous cell carcinoma (CESC) was downloaded from the TCGA database (up to May 26, 2017, https://portal.gdc.cancer.gov/), including individuals with clinical information. The following patient or lncRNA exclusion criteria were applied: 1) no complete tissue samples were present for data analysis; 2) histologic diagnosis was not CESC; 3) other malignancies apart from CESC were present; 4) The differentially expressed lncRNAs which were present in no more than 10% of the samples were eliminated. Overall, 289 CC patients were included in the present study providing 289 CC tissue samples and three adjacent normal tissue samples. The clinical data included staging information and outcomes of CESC patients. Processing this data did not violate the requirements of TCGA’s human protection and data access policies. (http://cancergenome.nih.gov/publications/publicationguidelines).

In addition, 31 CC patient tissue specimens (primary solid tumor and solid normal tissue) were collected from the Zhongda Hospital (Nanjing, China) of Southeast University, between 2016 and 2017, for qRT-PCR analysis. The tissues were snap-frozen in RNAlater (Ambion, Austin, TX, USA) after surgical resection and stored immediately in liquid nitrogen for subsequent total RNA extraction and analysis. These 31 patients (aged 23–64 years) were diagnosed with CC based on the histopathology and clinical history. All patients signed informed consent and this study was approved by the Zhongda Hospital Southeast University ethics committee.

### Identification of differentially expressed lncRNAs

The TCGA database provides normalized count data for RNA sequencing through the RNASeqV2 system, including lncRNA and mRNA expression profiles. The CESC level 3 lncRNA sequencing raw data were obtained through Illumina HiSeq 2000 RNA sequencing platforms (Illumina Inc., Hayward, CA, USA). Data were already normalized by the TCGA. The data of tumor CC tissues were already normalized to the adjacent normal tissues. RNA-Seq expression level read counts are normalized using two related methods: The Fragments per Kilobase of transcript per Million mapped reads (FPKM) and The upper quartile FPKM (FPKM-UQ). The differentially expressed RNAs included those upregulated and downregulated with fold changes >2 and <0.5, respectively, and adjusted false discovery rate at *P* < 0.05. To detect the differential expression of lncRNAs, samples were divided into three groups based on FIGO stage I, stage II and stages III–IV. The intersection of the lncRNAs was selected for further analysis. In addition, sequencing results of differentially expressed lncRNAs that showed no change in more than 10% of all samples were eliminated.

### Identification of the specific lncRNA prognostic signature

The expression level of each differentially expressed lncRNA was transformed by log2 to calculate the risk score. Subsequently, we further analyzed clinical features related to lncRNAs in CC. The univariate Cox proportional hazard model was used to analyze the effects of risk score and clinical features on the OS of CC patients (Bair & Tibshirani, 2004; Gao et al., 2016). The multivariate Cox proportional hazard model was used to evaluate the prognostic value of these OS-related lncRNAs. Based on the previously reported risk score model (Zhao & Sun, 2007), we constructed a prognosis-related risk score based on lncRNA expression levels. The formula is: Riskscore = explncRNA1 × β lncRNA1 + explncRNA2 × β lncRNA2+…+explncRNAn × β lncRNAn, where exp represents expression level and β represents the regression coefficient from the multivariate Cox regression model (Zeng et al., 2017). We used the median as the cutoff point in risk score. The CC patients were divided into high- and low-risk score group, respectively (Zhou et al., 2016). The receiver operating characteristic (ROC) curve analysis within 5 years was used to calculate the predictive value of the risk score for time-dependent outcomes (Heagerty, Lumley & Pepe, 2000).

### qRT-PCR verification of specific lncRNA expression in CC tissues and ROC curve analysis

We used qRT-PCR to analyze actual expression levels and validate the accuracy and reliability of the two lncRNAs in 31 newly diagnosed CC patients. All results were normalized to the control reference gene GAPDH. Following the standardized manufacturer’s protocol, total RNA was isolated from tissue samples using TRIzol reagent (Invitrogen, Carlsbad, CA, USA). RNA purity was determined using a NanoDrop 2000 spectrometer (Thermo Fisher Scientific, Waltham, MA, USA). RT reactions and qRT-PCR were both conducted according to the manufacturer’s protocol using the Reverse Transcription System and qPCR Master Mix kit (Promega, Madison, WI, USA), respectively. Step One Plus^TM^ PCR System (Applied Biosystems, Foster City, CA, USA) was used to detect the expression levels of lncRNAs. All the primers were produced by Generay Biotech Co., Ltd. (Shanghai, China). qRT-PCR results were calculated by the 2^−ΔΔCt^ method using the formulas ΔCt = Ct_ΔlncRNAs_ – Ct_ΔGAPDH_ and ΔΔCt = ΔCt_tumor tissues_ – ΔCt_adjacent non-tumor tissues_ (Wang, Chen & Liu, 2015). The ROC curve was used to evaluate the diagnostic value of the expression levels of the two lncRNAs. A *P* value of <0.05 was considered statistically significant.

### Cell culture and qRT-PCR verification of expression of two lncRNAs in cervical cancer cell lines

Three human CC cell lines (Hela, SiHa and C33-a), and an immortalized human cervical epithelial cell line (H8) were purchased from Shanghai Chuyu Biological Co., Ltd. The cells were cultured in 5% CO_2_ humidified atmosphere at 37 °C, using high-glucose Dulbecco’s modified Eagle’s medium (HyClone, South Logan, UT, USA) supplemented with 10% fetal bovine serum, 100 U/mL penicillin and 100 mg/mL streptomycin. qRT-PCR and total RNA extraction conditions, reagents and methods are the same as for tissues.

### Statistical analysis

Statistical analysis was performed by IBM SPSS Version 24.0. The final results are shown as means ± SD. Student’s *t*-tests were used to compare two groups of sequencing data. In all cases, *P* value of <0.05 was considered statistically significant. Fold change was used to analyze the statistical significance of results. Clinical parameters and risk scores were screened and compared using univariate and multivariate Cox regression models. In addition, based on the expression of lncRNA levels in CC patients, we performed ROC curve and area under curve (AUC) analyses to judge diagnostic value. R language was used as the main tool for generation of ROC curves (R Core Team, 2017).

## Results

### Identification of significantly differentially expressed lncRNAs and clinical characteristics of patients

**Figure 1:**
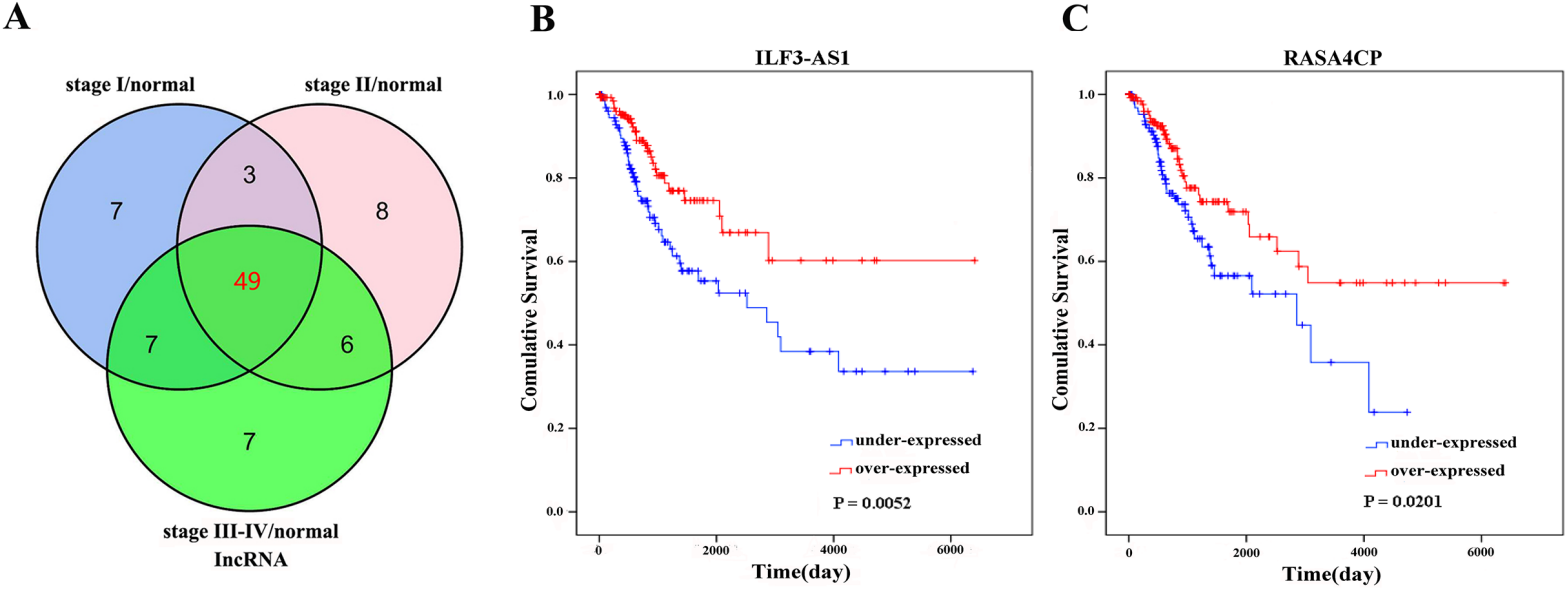
Forty-nine differentially expressed lncRNAs between FIGO stage I/Normal, FIGO stage II/Normal, FIGO stages III–IV/Normal. (A) Venn diagrams showing the number of common lncRNAs differentially expressed in different FIGO stages. (B) and (C) Two differentially expressed lncRNAs (ILF3-AS1 and RASA4CP). Kaplan–Meier curves showing the relationship between the lncRNAs and overall survival. The cases were divided into under- and over-expression groups.

There were 289 CC primary solid tumors and three solid normal tissues with clinical patient information obtained from TCGA which were included in the present study. The average age of patients was 47.31 ± 13.62 years. The OS time was 1045.70 ± 67.07 days, 71 patients died. Significant differentially expressed lncRNAs were identified based on the criteria of fold change >2 and <0.5 at *P* value <0.05. According to inclusion–exclusion criteria, we obtained 49 differentially expressed lncRNAs included in the intersection of the three groups’ analyzed results (Fig. 1A; Table 1). The available clinical features from the TCGA database are shown in Table 2.

**Table 1:** The common list of lncRNAs abnormally expressed in all FIGO stages in CECS.

lncRNA	Regulation	Fold-change*	*P*-value*
EMX2OS	Down	0.02	0.00000
CARMN	Down	0.02	0.00000
MIR4697HG	Down	0.04	0.00000
MIR100HG	Down	0.06	0.00001
MBNL1-AS1	Down	0.08	0.00000
HOXA11-AS	Down	0.10	0.00044
MEG3	Down	0.10	0.00020
LINC01140	Down	0.10	0.00000
LINC00341	Down	0.11	0.00000
A2M-AS1	Down	0.11	0.00000
TPTEP1	Down	0.12	0.00001
MIR99AHG	Down	0.12	0.00004
SERTAD4-AS1	Down	0.12	0.00004
NR2F1-AS1	Down	0.13	0.00044
SMIM10L2B	Down	0.16	0.00003
LINC00663	Down	0.19	0.00003
LINC00312	Down	0.21	0.00020
EPB41L4A-AS1	Down	0.21	0.00001
LINC00950	Down	0.25	0.00002
SNHG7	Down	0.26	0.00012
RASA4CP	Down	0.26	0.00004
GTF2IRD2P1	Down	0.26	0.00008
ATP1A1-AS1	Down	0.28	0.00012
ST7-AS1	Down	0.28	0.00168
ILF3-AS1	Down	0.30	0.00013
LINC00936	Down	0.30	0.00083
FAM66C	Down	0.30	0.00000
LOH12CR2	Down	0.31	0.00000
ACVR2B-AS1	Down	0.31	0.00033
AMZ2P1	Down	0.32	0.00028
FLJ10038	Down	0.33	0.00011
ZNF876P	Down	0.36	0.00032
FTX	Down	0.42	0.00137
EEF1A1P9	Down	0.43	0.00011
TOP1P1	Up	2.10	0.00062
EP400NL	Up	2.51	0.00012
LOC146880	Up	2.52	0.00014
OIP5-AS1	Up	3.10	0.00000
FBXO22-AS1	Up	3.71	0.00030
GOLGA2P10	Up	4.12	0.00149
GEMIN8P4	Up	4.72	0.00061
ASMTL-AS1	Up	4.91	0.00028
MST1P2	Up	5.21	0.00166
DDX12P	Up	5.21	0.00000
LINC00467	Up	5.74	0.00003
CDKN2B-AS1	Up	6.40	0.00009
GOLGA2P5	Up	6.69	0.00005
TMPO-AS1	Up	7.28	0.00000
MIR9-3HG	Up	49.93	0.00001

**Note:**

* Fold change >2 or <0.5, and *P* < 0.05.

**Table 2:** The available clinical characteristics of CESC cases and their relationship to overall survival.

Variables		Patient	Univariate analysis		Multivariate analysis	
		*N* (289)	HR (95% CI)	*P*	HR (95% CI)	*P*
Race	White	199	1 [reference]			
	Black	27	0.97 [0.46–2.05]	0.930		
	Asian	27	1.06 [0.38–2.96]	0.910		
	Others	2	7.39 [1–54.76]	0.050*		
Age	<45	130	1 [reference]			
	≥45	159	1.34 [0.82–2.18]	0.250		
BMI	Lean	12	1 [reference]			
	Normal	80	0.66 [0.23–1.95]	0.460		
	Overweight	155	0.45 [0.16–1.3]	0.140		
HPV	Low	265	1 [reference]			
	High	2	3.38 [0.46–24.6]	0.230		
Tobacco	Non-smoker	141	1 [reference]			
	Current smoker	110	1.33 [0.81–2.18]	0.260		
Clinical stage	Stage i	159	1 [reference]		1 [reference]	
	Stage ii	68	0.81 [0.41–1.6]	0.550	1.66 [0.77–3.58]	0.196
	Stage iii	44	1.28 [0.63–2.58]	0.490	0.39 [0.39–4.53]	0.648
	Stage iv	18	4.41 [2.33–8.32]	<0.001*	3.70 [1.73–7.90]	<0.001*
T stage	t1+t2	206	1 [reference]			
	t3+t4+tx	43	3.58 [2.01–6.35]	<0.001*		
N stage	n0	130	1 [reference]			
	n1	57	0.25 [0.13–0.5]	<0.001*		
	nx	62	0.71 [0.36–1.38]	0.310		
M stage	m0	112	1 [reference]			
	m1	9	4.11 [1.38–12.23]	0.010*		
	mx	124	1.93 [1.08–3.44]	0.030*		
Neoplasm cancer	Tumor free	189	1 [reference]		1 [reference]	
	With tumor	78	21.27 [11.11–40.72]	<0.001*	29.27 [12.54–68.30]	<0.001*
Menopause	Pre	121	1 [reference]			
	Post	75	1.54 [0.54–4.43]	0.420		
	Peri	25	1.65 [0.57–4.82]	0.360		
RISK	Low	144	1 [reference]		1 [reference]	
	High	145	2.48 [1.5–4.12]	<0.001*	2.60 [1.42–4.95]	0.002*

**Note:**

HR, hazard ratio; CI, confidence interval.

* *P* < 0.05.

### Construction of an lncRNA signature significantly associated with prognostic features

**Figure 2:**
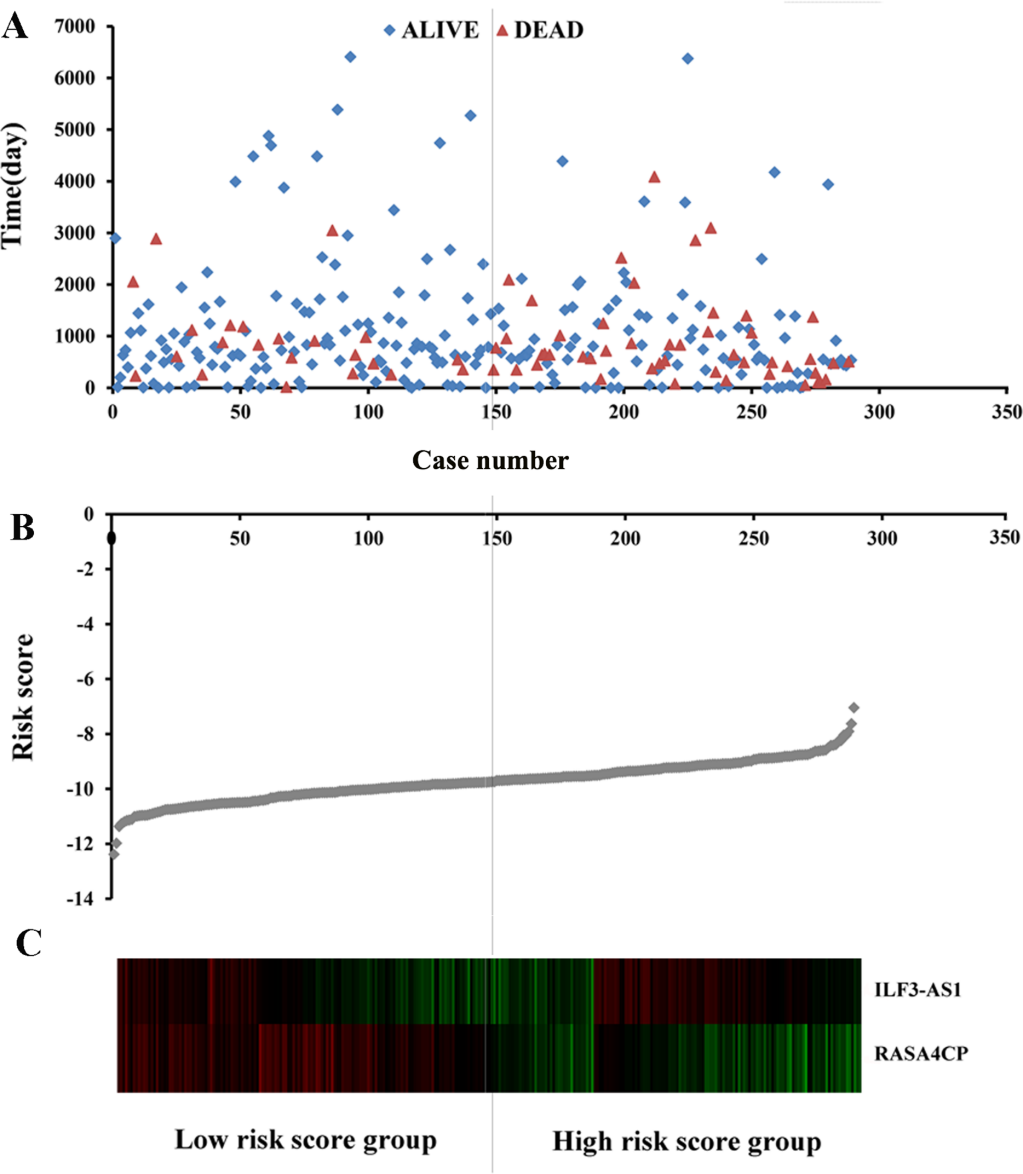
Risk score analysis of the differentially expressed lncRNA signature of cervical cancer. (A) Survival status and duration of cases. (B) Risk score of lncRNA signature; (C) Heatmap displaying low- and high-risk score groups for the two lncRNAs. The gray line represents the cut-off values for the high- and low-risk score groups.

**Figure 3:**
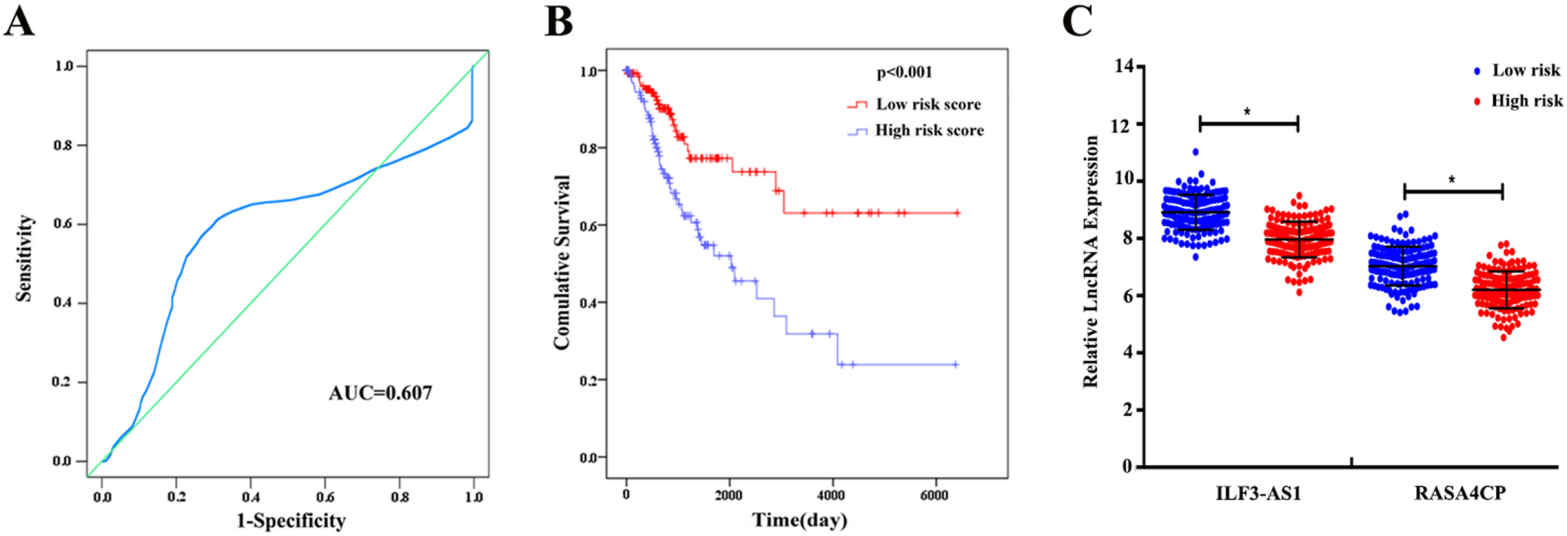
The two differentially expressed lncRNA signature of cervical cancer for the outcome based on TCGA. (A) The two-lncRNA signature is shown by the time-dependent ROC curve for predicting 5-year survival. (B) The Kaplan–Meier curve of the risk score for the overall survival. (C) The expression level of lncRNAs between the low-risk and high-risk groups. *P < 0.05.

The 49 differentially expressed specific lncRNAs were further analyzed. We analyzed race, age, body mass index, human papillomavirus, tobacco use, clinical stage, tumor-node-metastasis staging system, tumor status and menopause in the TCGA database. In the univariate Cox proportional hazard model, four of the 49 differential expressed lncRNAs had important prognostic value (*P* < 0.05; Table 3). Further analysis using multivariate Cox regression showed that only two lncRNAs were important and independent biomarkers for OS in CC patients: ILF3-AS1 and RASA4CP (*P* < 0.05; Table 3). These two specific lncRNAs were positively correlated with OS (log-rank *P* < 0.05; Figs. 1B and 1C). Using ROC curve analysis, the AUC values for ILF3-AS1 and RASA4CP were calculated as 0.963 and 0.988, respectively (*P* < 0.05; Fig. S1). The AUC of these two lncRNAs together was 0.991, which was higher than that of each lncRNA taken alone (*P* < 0.05). We next built a risk score for predicting the prognostic value. The formula was Risk score = expILF3-AS1 × (−0.703) + expRASA4CP × (−0.576). The 289 patients were divided into low-risk (*n* = 144) and high-risk (*n* = 145) groups (Fig. 2). The survival time of patients in the low-risk score group was 1152.48 ± 104.27 days compared to 939.66 ± 83.98 days in the high-risk score group. The risk score largely predicted 5-year survival of CC patients, as the AUC upon ROC curve analysis was 0.607 (Fig. 3A). Furthermore, Kaplan–Meier curves showed that low-risk group was positively correlated with OS, and the survival time of patients in the low-risk group was longer than that of the high-risk group (*P* < 0.001; Fig. 3B).

**Table 3:** Prognostic value of the differentially expressed lncRNAs by univariate and multivariate Cox regression analysis.

Variables	Estimate	StdErr	ChiSq	*P*	HR (95% CI)
Univariate Cox					
ILF3-AS1	−0.692	0.252	7.510	0.006*	0.501 [0.305–0.821]
RASA4CP	−0.573	0.245	5.271	0.022*	0.570 [0.352–0.921]
LINC00341	−0.483	0.242	3.975	0.046*	0.617 [0.384–0.992]
AMZ2P1	−0.553	0.248	4.994	0.025*	0.575 [0.354–0.934]
Multivariate Cox					
ILF3-AS1	−0.703	0.253	7.742	0.005*	0.302 [0.302–0.821]
RASA4CP	−0.576	0.245	5.514	0.019*	0.347 [0.347–0.909]

**Note:**

Estimate: β coefficient; HR, hazard ratio; CI, confidence interval.

* *P* < 0.05.

### The prognostic value of the two-lncRNA signature and other clinical features

Based on the results of univariate Cox proportional hazard model analysis, some of the clinical features may predict poorer survival outcomes of CC patients (Table 2). In addition, we further analyzed by the multivariate Cox proportional hazard model tumor status (*P* < 0.001) and the risk score (*P* = 0.002), which were determined to be two independent prognostic factors of CC. The Kaplan–Meier curves of the aforementioned clinical features are shown in Fig. S2. The results showed that clinical stage (*P* = 0.001), T stage (*P* < 0.001), N stage (*P* < 0.001), M stage (*P* = 0.011) and tumor status (*P* < 0.001) were related to OS. We also evaluated the relationship between the risk score based on the differentially expressed two-lncRNA signature and the clinical features, and the risk score showed prognostic value for predicting the status of T stage (AUC = 0.607, *P* = 0.028; Fig. S3). The expression levels of two differentially expressed lncRNAs in the low and high score groups in TCGA are shown in Fig. 3C. The results revealed that the expression level of lncRNA RASA4CP was significantly different between the low-risk and high-risk groups (*P* < 0.05).

### qRT-PCR verification of the expression level of two lncRNAs in tissues and ROC curve analysis

**Figure 4:**
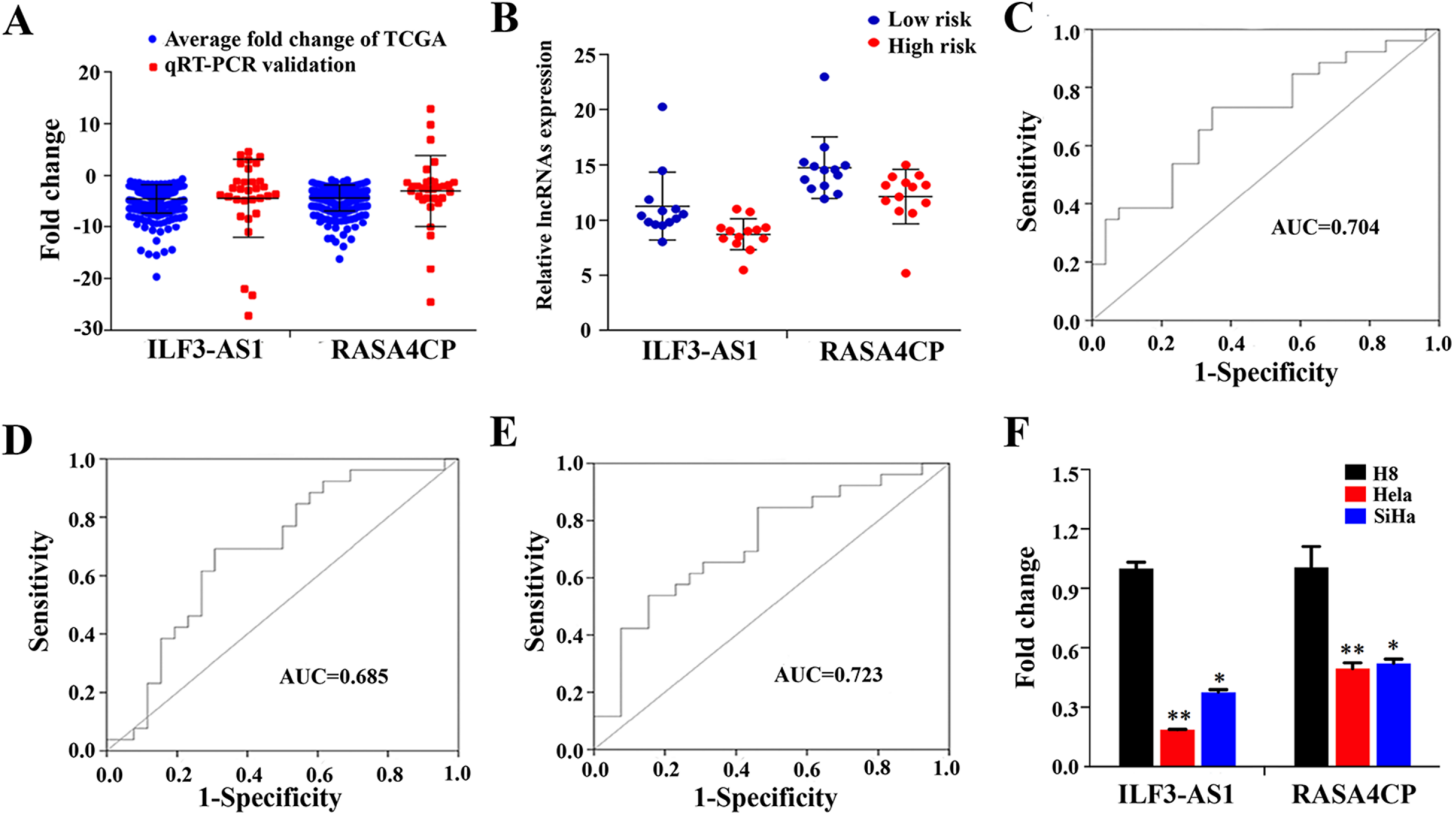
Analysis of expression of ILF3-AS1 and RASA4CP in clinical samples with qRT-PCR. (A) Quantitative RT-PCR validation of differentially expressed two lncRNAs (Comparison of fold change (2^−ΔΔCt^) of lncRNAs between TCGA results and qRT-PCR results). (B) The expression level of lncRNAs between the low-risk and high-risk groups based on qRT-PCR. (C, D and E) ROC curve analysis of specific two lncRNAs with relative expression level. (F) qRT-PCR validation of differentially expressed two lncRNAs (comparison of fold change (2^−ΔΔCt^) of lncRNAs between three cervical cancer cell lines results and one immortalized cervical epithelial cell line results).

To confirm the expression levels of ILF3-AS1 and RASA4CP in CC, we analyzed their actual expression levels in 31 pairs of newly diagnosed CC clinical samples by qRT-PCR (Table S1). The qRT-PCR results confirmed that the expression of the ILF3-AS1 and RASA4CP was downregulated, consistent with the TCGA results (Fig. 4A). The actual expression levels of the two lncRNAs in the low-risk score and high-risk score groups in the clinical sample are shown in Fig. 4B. To evaluate the specific diagnostic potential of ILF3-AS1 and RASA4CP, ROC curve analysis was performed. AUC values were 0.704 and 0.685 for ILF3-AS1 and RASA4CP, respectively, (*P* < 0.05; Figs. 4C and 4D). This indicates that each lncRNA could be an important biomarker for diagnosis of CC. However, the AUC value for both lncRNAs was 0.723, higher than that any single lncRNA alone (*P* < 0.05; Fig. 4E), suggesting improved diagnostic efficiency using the two-lncRNA signature.

### qRT-PCR verification of the two lncRNAs’ expression in cervical cancer cell lines

To detect ILF3-AS1 and RASA4CP expression in CC, we further examined gene expression pattern from three CC cell lines by qRT-PCR. Compared to the immortalized cervical epithelial cell line H8, expression of ILF3-AS1 and RASA4CP was downregulated in the human CC cell lines HeLa and SiHa, respectively. The expression pattern of the two lncRNAs is consistent with the TCGA results (Fig. 4F).

## Discussion

With vaccination and early tumor screening programs, the incidence and mortality of CC have declined in recent decades. However, the incidence of CC in developing countries still remains high (Arbyn et al., 2011; Siegel, Naishadham & Jemal, 2012b). The prognosis for CC patients would be improved if tumor development could be predicted in preclinical diagnosis. In recent years, non-coding RNAs in CC have been widely investigated (Chen et al., 2017b; Yang et al., 2016b), but most studies have mainly focused on the relationship and expression of mRNAs, miRNAs, genes and proteins (Liao et al., 2014; Liao et al., 2017; Liu et al., 2013) in CC. At present, there is still a lack of specific and effective biomarkers for use in diagnosis, clinical therapy and prognostic applications. Therefore, there is an urgent need to identify potential and reliable prognostic biomarkers to predict CC outcomes.

Based on the large-scale datasets provided by the TCGA public database, a number of studies have evaluated the prognostic value of lncRNAs in various cancer types such as gastric cancer, lung cancer, colorectal cancer and breast cancer (Nie et al., 2017; Song et al., 2017; Xie et al., 2017; Zheng et al., 2017). A new study evaluating the prognostic value of miRNAs in CC (Ying et al., 2017) assessed the potential use of a three-miRNA (miR-3154, miR-7-3 and miR-600) expression signature as well as individual miRNAs as prognostic biomarkers of CC. Similarly, another study using Cox regression analysis showed that a three-miRNA signature (miR-200c, miR-145 and miR-218-1) signature could be used as an independent prognostic factor in CC (Liang, Li & Wang, 2017). This analysis used the TCGA database; however, the relationships between prognosis and differentially expressed lncRNAs in CC patients using large-scale samples have not yet been comprehensively analyzed.

In our study, we defined lncRNAs that were significantly associated with survival by screening differentially expressed lncRNAs from the TCGA database and using a large sample of CC patients. First, based on RNA sequencing data from TCGA (*P* < 0.05), the Cox regression model was used on 49 differentially expressed lncRNAs from 289 CC patients. Two lncRNAs (ILF3-AS1 and RASA4CP) were identified. Subsequently, we calculated the risk score by combining the expression of these two lncRNAs and investigated whether this two-lncRNA signature could be used to independently predict OS in CC patients. Our results have shown that the single marker efficacy was limited, but multiple markers may provide more effective information for the prediction of cancer patients’ prognoses. In the present study we compared the clinical features and sequencing data to investigate the relationship between the two lncRNAs and CC patients’ survival by performing a risk score assessment. To our knowledge, this study is the first to combine risk scores with information about lncRNAs from the TCGA data to assess the survival and prognosis of CC patients.

To date, one report has shown that in melanoma, upregulated ILF3-AS1 promotes cell migration, invasion and proliferation by negatively regulating miR-200b/a/429, which implies that ILF3-AS1 may be a potential prognostic biomarker and therapeutic target for melanoma (Chen et al., 2017a). However, the role of this lncRNA in the onset of CC has not been reported. Similarly, the role of RASA4CP in cancer has not been explored. Dysregulation in signaling pathways may play a crucial role in CC pathogenesis and progression. In the future, we intend to further analyze the signaling pathways associated with these lncRNAs by performing mechanistic research.

We also performed cross-validation of our findings, with qRT-PCR of which the results were in agreement with those from TCGA. We tried to verify the expression of ILF3-AS1 and RASA4CP in a Chinese population sample using risk scores, but the results were not statistically significant, probably because of the small sample size. However, we could see that the qRT-PCR results had the same trend as the TCGA data. We used ROC curve analysis to determine the sensitivity and specificity of ILF3-AS1 and RASA4CP as key lncRNAs in the detection of CC. Each of the two lncRNAs showed diagnostic value when considered alone, but more importantly, the AUC of the combination of two lncRNAs was 0.723 (*P* < 0.05). This value was greater than that of each lncRNA alone, suggesting that the combination of these two lncRNAs could improve the diagnostic efficacy in CC. While we believe our findings have important clinical value, there are still some limitations which should nonetheless be considered. First, our findings need validation over a longer follow-up time. Second, additional data from TCGA combined with further molecular investigations and more clinical samples are needed to verify our findings. Finally, the function of ILF3-AS1 and RASA4CP in CC need to be examined in future studies.

## Conclusion

In conclusion, this is the first time to our knowledge that the TCGA public database has been used to identify lncRNAs that are significantly associated with prognosis of CC. The two-lncRNA signature of ILF3-AS1 and RASA4CP may serve as a potential independent biomarker for predicting CC prognosis. However, future studies are necessary to further explore the function and mechanism of these lncRNAs in CC.

## Supplemental Information

10.7717/peerj.6761/supp-1 Supplemental Information 1 LncRNA sequencing data of TCGA.

10.7717/peerj.6761/supp-2 Supplemental Information 2 Fig. S1. ROC curves of the two lncRNAs (ILF3-AS1 and RASA4CP) to distinguish cervical cancer tissue from adjacent normal tissues in TCGA.

10.7717/peerj.6761/supp-3 Supplemental Information 3 Fig. S2. The prognostic value of different clinical features for overall survival of cervical cancer patients in TCGA.

10.7717/peerj.6761/supp-4 Supplemental Information 4 Fig. S3. The predictive value of the risk score for clinical features. ROC curve is predicting different clinical features in TCGA.

10.7717/peerj.6761/supp-5 Supplemental Information 5 Table S1. Relative expression of lncRNAs in 31 pairs of cervical cancer tumor and non-tumor tissues.

10.7717/peerj.6761/supp-6 Supplemental Information 6 Table S2. The protein-protein interaction network of co-expressed genes. Gene name and Pearson |R|

## Additional Information and Declarations

### Data Availability

The following information was supplied regarding data availability:

The raw data are provided in the Supplemental Files.
