# Supplementary figures and images for: Integrated analysis of two-lncRNA signature as a potential prognostic biomarker in cervical cancer: a study based on public database
Source: PeerJ. 2019 Apr 22;7:e6761. doi: 10.7717/peerj.6761 (PMC6482937; doi:10.7717/peerj.6761)

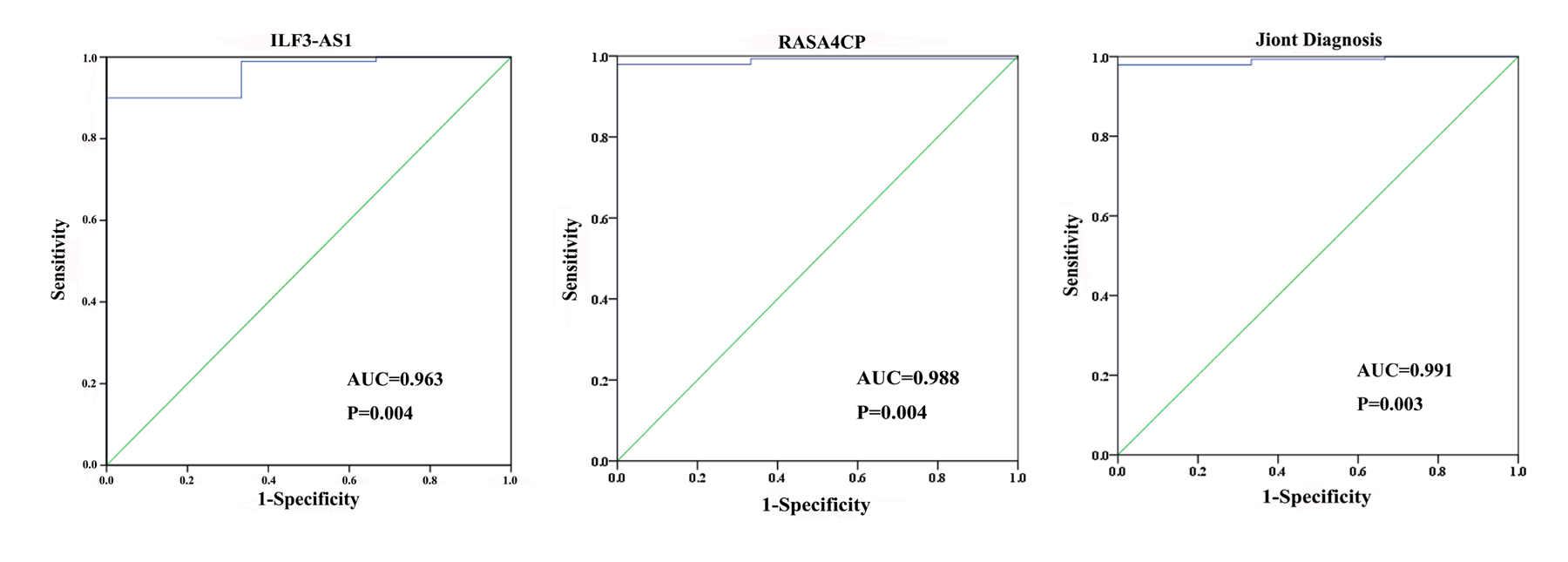

Supplement: Supplemental Information 2 [file peerj-07-6761-s002.png]

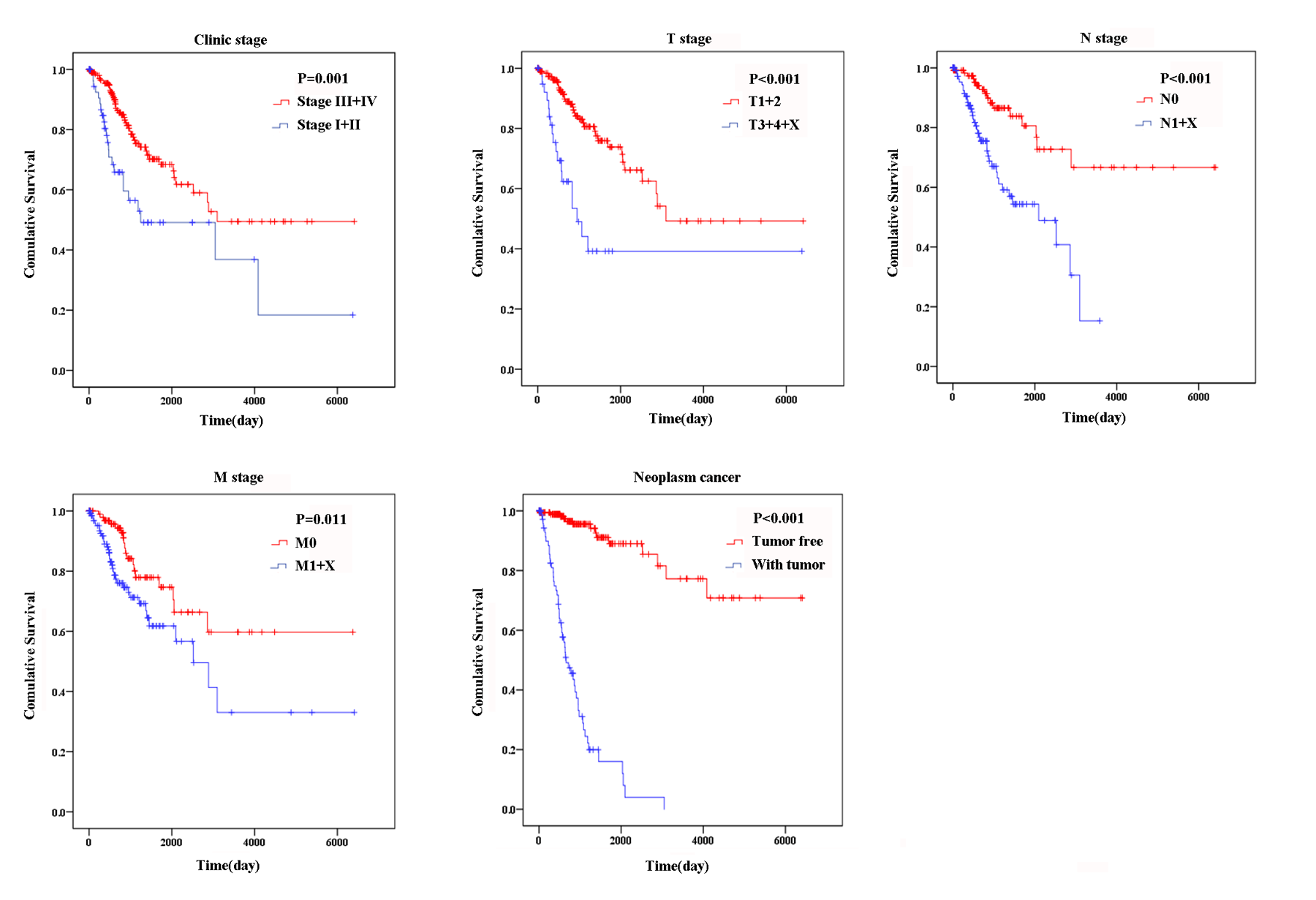

Supplement: Supplemental Information 3 [file peerj-07-6761-s003.png]

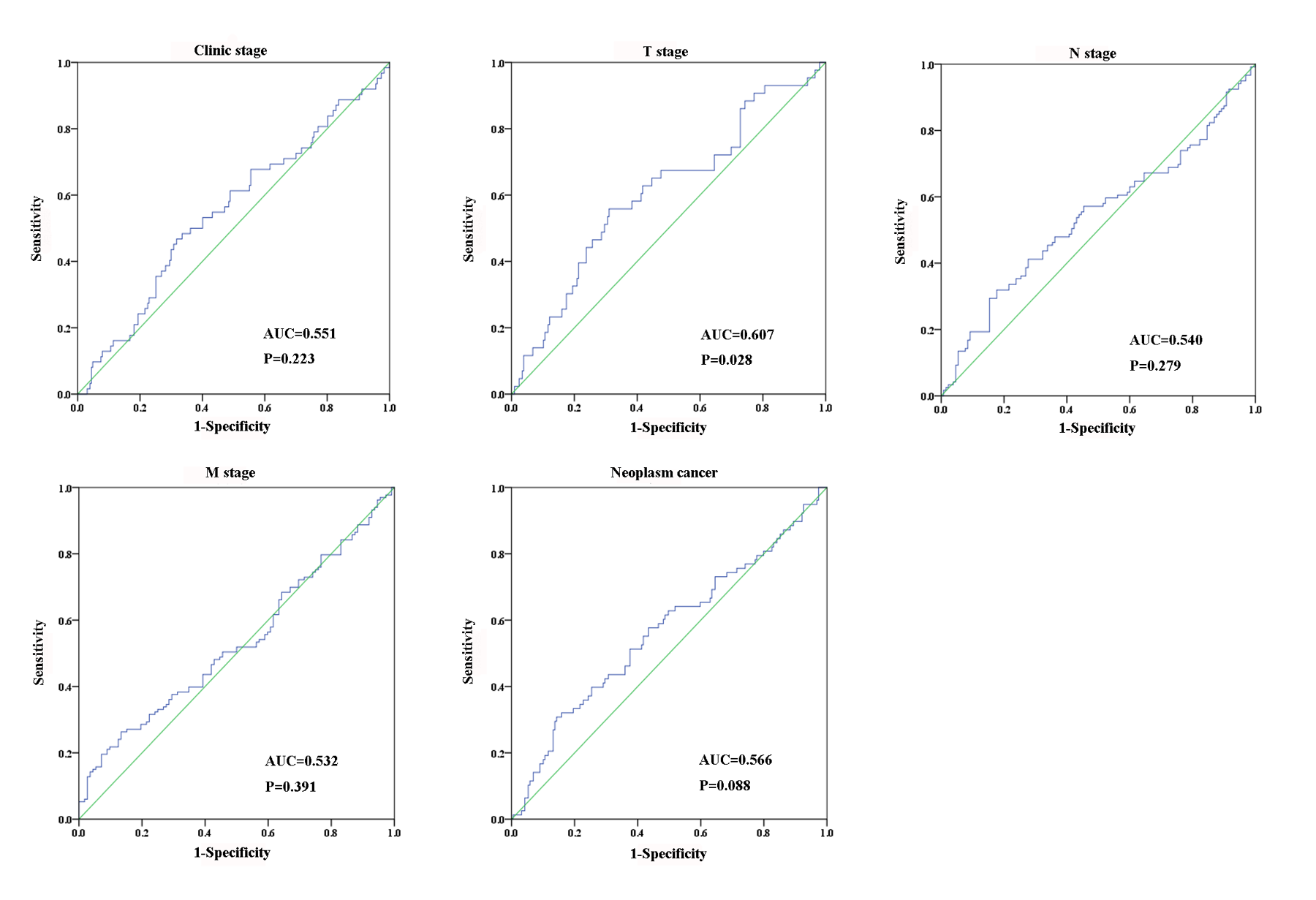

Supplement: Supplemental Information 4 — ROC curve is predicting different clinical features in TCGA. [file peerj-07-6761-s004.png]
