# Supplementary material for: Integrated analysis of two-lncRNA signature as a potential prognostic biomarker in cervical cancer: a study based on public database
Source: PeerJ. 2019 Apr 22;7:e6761. doi: 10.7717/peerj.6761 (PMC6482937; doi:10.7717/peerj.6761)
Supplement: Supplemental Information 5 [file peerj-07-6761-s005.docx]

**Table 1.** Relative expression of lncRNAs in 31 pairs of cervical cancer tumor and non-tumor tissues

| **Gene symbol** | **Type** | **Group** | **Mean ± SD of ΔCt** | **ΔΔCt^a^ (mean ± SD)** | **2^-ΔΔCt^** | **P-value** | **t-value** |
| --- | --- | --- | --- | --- | --- | --- | --- |
| **ILF3-AS1** | LncRNA | Tumor tissues | 9.828±2.665 | 1.283±1.993 | -4.551 | 0.002* | 3.468 |
|  |  | Adjacent non-tumor tissues | 8.545±2.604 |  |  |  |  |
| **RASA4CP** | LncRNA | Tumor tissues | 13.510±2.897 | 1.431±3.018 | -19.985 | 0.016* | 2.553 |
|  |  | Adjacent non-tumor tissues | 12.079±2.295 |  |  |  |  |
| a: ΔCt = Ct _target gene_ – Ct _GAPDH_; ΔΔCt = ΔCt _tumor tissues_ –ΔCt _Adjacent non-tumor tissues_. b: P<0.05. | | | | | | | |
